# Feeding Marine Polysaccharides to Alleviate the Negative Effects Associated with Weaning in Pigs

**DOI:** 10.3390/ani11092644

**Published:** 2021-09-09

**Authors:** John V. O’Doherty, Brigkita Venardou, Ruth Rattigan, Torres Sweeney

**Affiliations:** 1 School of Agriculture and Food Science, University College Dublin, Belfield, D04 V1W8 Dublin 4, Ireland; ruth.rattigan@ucdconnect.ie; 2 School of Veterinary Medicine, University College Dublin, Belfield, D04 V1W8 Dublin 4, Ireland; brigkita.venardou@ucdconnect.ie (B.V.); torres.sweeney@ucd.ie (T.S.)

**Keywords:** pig, weaning, marine polysaccharides, dietary supplement

## Abstract

**Simple Summary:**

Weaning is the most crucial event in commercial pig farms. It involves complex dietary, social, and environmental stresses that interrupt gut development in the pig. These stresses have been controlled with in-feed prophylactic antibiotics and dietary minerals. These strategies are under scrutiny because of their role with antimicrobial resistance and environmental contamination. There is an urgency to find alternative dietary supplements that can support growth and prevent diarrhoea in the weaned pig. Marine macroalgae and organisms offer an interesting source of novel bio-actives. The supplementation of intact (whole) seaweed has not been successful in the immediate post-weaned pig diet, probably due to negative interaction between the constituents on digestive health and performance. Supplementation with the purest forms of laminarin and fucoidan extracted from macroalgae and chitin derivatives appear to have the most benefit in terms of improvements in gastrointestinal health. This is due to their prebiotic, antibacterial, anti-oxidant, and immunomodulatory properties. The extraction methodologies and conditions used to extract these polysaccharides are also an important contributing factor to the biological properties of these polysaccharides. This review focuses on the feeding of laminarin, fucoidan, and chitin derivatives as suitable substitutes for in-feed prophylactic antibiotics and minerals.

**Abstract:**

In young pigs, the challenge of weaning frequently leads to dysbiosis. This predisposes pigs to intestinal infection such as post-weaning diarrhoea (PWD). Dietary interventions to reduce PWD have centred on dietary inclusion of antibiotic growth promoters (AGP) and antimicrobials in pig diets, or high concentrations of zinc oxide. These interventions are under scrutiny because of their role in promoting multidrug resistant bacteria and the accumulation of minerals in the environment. There are significant efforts being made to identify natural alternatives. Marine polysaccharides, such as laminarin and fucoidan from macroalgae and chitosan and chito-oligosaccharides from chitin, are an interesting group of marine dietary supplements, due to their prebiotic, antibacterial, anti-oxidant, and immunomodulatory activities. However, natural variability exists in the quantity, structure, and bioactivity of these polysaccharides between different macroalgae species and harvest seasons, while the wide range of available extraction methodologies and conditions results in further variation. This review will discuss the development of the gastrointestinal tract in the pig during the post-weaning period and how feeding marine polysaccharides in both the maternal and the post-weaned pig diet, can be used to alleviate the negative effects associated with weaning.

## 1. Introduction

Weaning is the most crucial event in commercial pig farms in terms of animal productivity and health. The newly weaned pig not only transits from milk to a solid and more complex diet, but is also subjected to additional stressors including separation from sow and littermates, co-mingling with unknown pigs, adaptation to new environmental settings, and increased pathogen exposure [1]. All these stressors result in reduced feed intake, lasting up to 48 h post-weaning, which is the main driver of the observed gastrointestinal dysfunction, poor performance, and post-weaning diarrhoea (PWD) [2,3]. Traditional measures to reduce weaning associated intestinal dysfunction have centred on dietary inclusion of antibiotic growth promoters (AGP) in weaning pig diets [4], or high concentrations of dietary minerals in the form of zinc oxide at doses well above nutritional requirements. The direct purpose of these additives is to suppress the growth of pathogenic bacteria such as *Escherichia coli* and *Salmonella enterica* subsp. *enterica* serotypes. However, owing to the possible contribution of in-feed antibiotics to the development of antibiotic resistant strains of bacteria [5], the European Union implemented a full ban on AGP usage in livestock diets in January 2006. Zinc oxide (ZnO) was a successful alternative to deal with the negative impact of weaning on growth and gastrointestinal dysfunction (including dysbiosis) in pigs [6], but ZnO will also be banned in the EU by 2022 due to its association with environmental contamination and antimicrobial resistance ((Commission Implementing Decision of 26.6.2017, C(2017) 4529 Final). Furthermore, the use of antimicrobials in farm animals will be subjected to additional restrictions in the EU from 2022 (Regulations (EU) No. 2019/6 and No. 2019/4). Thus, there is an increasing urgency for alternative dietary supplements that can support growth and gastrointestinal health and functionality in the post-weaned pig.

Marine polysaccharides from macroalgae and chitin provide an interesting source of novel bio-actives and are interesting group of natural dietary supplements for use in pig nutrition due to their prebiotic, antibacterial, and immunomodulatory activities [7,8]. Hence, they offer great potential as preventatives and prophylactics in pig diets. This review will discuss the development of the intestinal tract and the factors that influence intestinal health on the pig during post-weaning period. It will also explore the potential for marine polysaccharides in maternal and post-weaning diets to alleviate the negative impact of weaning on growth and health.

## 2. The Negative Biological Effects Associated with Weaning

Weaning is a critical period in pig husbandry. In the wild, pigs naturally wean at 10–12 weeks of age, which coincides with the almost complete development and maturation of the gastrointestinal tract (GIT); in contrast, commercial weaning occurs at 2–4 weeks of age. Commercial weaning induces transient alternations to the gastrointestinal tract (GIT). These morphological and physiological changes are most likely driven by the post-weaning reduction in feed intake. As feed intake resumes, the GIT undergoes a period of intestinal maturation [9]. The villi and crypts that line the epithelium of the small intestine are essential for the digestive and absorptive processes [10]. Dietary composition has marginal effects on the small intestinal morphology of weaned pigs, with the level of feed intake found to be the most important determinant of mucosal function and integrity [11]. Food deprivation leads to a lack of luminal stimulation. This results in a rapid decrease in villous height [10]. Villous height is at its lowest after 2–5 days post-weaning, resulting in a reduced ability to absorb nutrients [12]. Villous height starts to recover in feed deprived piglets 4 days after feeding is restarted and can take more than 10 days to completely recover [13]. The villus surface area is also altered in the post-weaning period. Pre-weaning, villi are dense and finger-like, while the weaning transition changes the villi into predominantly smooth, compacted, and tongue-shaped villi [14]. As well as the intestinal morphology being affected by weaning, gastrointestinal functionality is also impaired as indicated by the reduction in brush border enzymes such as lactase, sucrase, and peptidases, and the disturbances in nutrient absorption and electrolyte secretion with the latter also contributing to the weaning-associated diarrhoea [12,13,15]. The resulting maldigestion and malabsorption leads to the weight loss observed during the first 4–5 days post-weaning [16,17].

A compromised intestinal barrier characterised by increased paracellular permeability, reduced transepithelial resistance, and reduced gene expression of tight junction proteins is additionally observed at the immediate post-weaning period and may lead to overstimulation of the immune system due to the increased presence of dietary and microbial antigens [5,16,18]. The activation of the immune system further contributes to the reduced intestinal barrier function and diarrhoea in newly weaned pigs. Several studies have reported infiltration of immune cells such as lymphocytes, macrophages, and mast cells in the lamina propria [2,5], increased expression of genes encoding for inflammatory cytokines such as tumour necrosis factor (*TNF*), interferon gamma (*INFG*), and interleukins *IL1B* and *IL6* [18,19], and activation of several pathways associated with immune responses [17] in the small and large intestine of pigs in the immediate post-weaning period.

The composition of the GIT microbiota is also altered in response to the weaning stress, diet alteration, reduced feed intake, and gastrointestinal dysfunction. Several studies have investigated the weaning-induced compositional and functional changes in the GIT microbiota of pigs [20,21,22,23,24]. *Lactobacillus* spp. are amongst the intestinal bacterial populations that are frequently monitored during the post-weaning period due to their high abundance in pigs and known beneficial effects. A significant reduction of this population, as well as shifts of the dominant strains, has been observed in the ileum of pigs post-weaning [25,26]. The decrease in the *Lactobacillus* spp. is transient, as seen in the ileum and faeces of weaned pigs and is followed by restoration or even an increase in its numbers and dominance of strains that utilise complex carbohydrates [20,21,24,26,27]. *Enterobacteriaceae* is an important indicator of dysbiosis in the faeces of newly weaned pigs, as an increase in the counts of this bacterial family was associated with higher incidence of diarrhoea [28]. Nevertheless, the increase in *Enterobacteriaceae* relative abundance is transient under normal circumstances, as this bacterial population and its members (*Escherichia*/*Shigella*) are minor constituents of the maturing GIT microbiota [21,22,26,27]. The reduction in *Bacteroides* spp. and increase in *Prevotella* spp. is another common change in the faecal microbiota of weaned pigs that is probably associated with the transition from milk mono- and oligo-saccharides to plant-derived polysaccharides [20,21,23]. Weaning-induced gastrointestinal dysbiosis is considered a key contributor to the development of diarrhoea and predisposes pigs to PWD [29]. The most common causative agent of PWD is the α-haemolytic Gram-negative enterotoxigenic *E. coli* (ETEC) that colonises the epithelium of the small intestine via F4 (ab, ac, ad) and F18 (ab, ac) fimbriae and non-fimbrial AIDA (adhesin involved in diffuse adhesion) [30,31]. Several studies have investigated the role of the weaned GIT microbiota in the development of diarrhoea and PWD. A study carried out by Dou et.al. [28] identified *Prevotelleaceae*, *Lactobacillaceae*, *Lachnospiraceae*, and *Ruminococcaceae* as faecal indicators of reduced diarrhoea incidence post-weaning. Furthermore, reduced Bacteroidetes:Firmicutes ratio and *Prevotella* spp. relative abundance and increases in *Escherichia*/*Shigella* and *Lactococcus* genera in jejunum and faeces were considered indicative of GIT dysbiosis in diarrhoeal weaned pigs challenged with ETEC, whereas *Lactobacillus* genus was deemed beneficial for recovering from PWD [32].

## 3. Traditional and Alternative Dietary Interventions

Dietary interventions are one strategy with which to prevent or alleviate dysbiosis and its associated impact on the growth and health of pigs. A diverse range of feed additives have been studied as preventatives and prophylactics in pig diets. An array of natural compounds have been investigated as alternative strategies to AGPs and ZnO such as yeast β-glucans [33,34], mannan-oligosaccharides [35], prebiotics such as galacto-oligosaccharides [36], organic acids [37,38], probiotics [39], spray dried plasma proteins [40], exogenous feed enzymes [41], and essential oils [42]. These compounds can support the microbial composition, health, and growth performance of pigs. However, there is only a limited number of compounds that result in a similar improvement in growth performance and reduced the occurrence of diarrhoea compared to in-feed AGP or ZnO. Therefore, there is still a need to identify natural bio-actives with growth promoting and immunomodulatory properties as suitable substitutes to AGPs and ZnO. It is also critical to explore the underlying mechanisms when evaluating the functional properties of feed ingredients and feed additives [43]. Key components of GIT function that should be considered include absorptive capacity (villi architecture and nutrient transporters expression), digestive capacity (activity of pancreatic and brush-border enzymes), physical and chemical barriers, microbial load, microbial diversity, and immune function.

## 4. Marine Polysaccharides

Marine macroalgae, broadly classified into brown, red, and green seaweeds, are a major source of novel bio-actives with potential benefits on animal health. While they consist of ≥94% water, they also contain varying concentrations of non-digestible polysaccharides, polyphenols, minerals, vitamins, proteins, and lipids [44]. Of particular interest are the non-digestible polysaccharides of brown seaweeds, namely alginate and fucoidan which, along with cellulose, are structural components of the algal cell wall, while laminarin and mannitol are located in the cytoplasm [44,45,46]. Feeding intact or whole macroalgae has attracted considerable interest in recent years as potential substitutes for AGP and ZnO to maintain performance and health in weaner pigs, due to their prebiotic, antibacterial, antioxidative, and immunomodulatory activities [47,48].

The supplementation with crude seaweed extracts containing both laminarin and fucoidan have been shown to be effective in post-weaned pig diets [49,50,51,52], however, the supplementation of intact seaweed has been less successful in the immediate post-weaned pig diet, as presented in Table 1. In a recent large commercial experiment in Denmark, Satessa et al. [53] could not obtain any positive effects of intact macroalgae on piglet health and performance. Previous studies with intact brown macroalgae also reported similar results in weaned pigs [54,55] or reduced performance when fed to finishing pigs [56]. The application of the intact macroalgae in a dry meal, means that the nutritional value of the final product is dependent on the seaweed variety, season of harvest, geographic location, and environmental and climatic conditions, all of which influence chemical composition [57,58,59,60]. The extraction methodologies and conditions used to extract polysaccharides (i.e., combination of parameters such as solvent, pH, temperature, time, solvent to seaweed ratio) are also an important contributing factor to the quantitative, structural, and functional variability of seaweed polysaccharides [58,59,61].

Chitin is a natural polysaccharide found in the exoskeletons of arthropods. Chitosan is formed by partial deacetylation of chitin under alkaline conditions or by enzymatic hydrolysis. Chitosan has exhibited antimicrobial activities against many bacteria, fungi, and yeasts, with a high killing rate for both gram-positive and gram-negative bacteria and low toxicity towards mammalian cells, indicating its suitability as an antimicrobial supplement [62]. The antimicrobial activities of chitosan are dependent on several factors including pH, the species of the microorganism, pKa, molecular weight, degree of deacetylation, and the presence or absence of metal cations [63]. This review will focus on the feeding of laminarin, fucoidan, chitosan, and chitosan derivatives and their ability to alter the composition of the GIT microbiota, inhibit intestinal pathogens, modulate the immune system, and enhance performance and health in the post-weaned pig.

**Table 1:** Effect of seaweed supplement on growth performae, diarrhoea scores and parameters of gastro intestinal functionality.

Pig Age	Dietary Supplement	Dose	Time and Duration of Supplementation	Effect on Growth Performance and Diarrhoea Scores	Effect on Parameters of GIT Functionality and Health	Ref.
Weaned pigs						
24-day-old	Laminarin (*Laminaria* spp.) Fucoidan (*Laminaria* spp.) Laminarin + Fucoidan	300 mg/kg 240 mg/kg 300 mg/kg + 240 mg/kg	After weaning for 21 days	+ ADG and G:F in pigs fed laminarin-supplemented diets + ADG in pigs fed with diet supplemented solely with fucoidan (interaction) − diarrhoea score in pigs fed laminarin-supplemented diets	− faecal *E. coli* in pigs fed laminarin-supplemented diets + faecal *Lactobacillus* spp. in pigs fed with diet supplemented solely with fucoidan (interaction)	[49]
24-day-old	Laminarin (*Laminaria* spp.) Fucoidan (*Laminaria* spp.) Laminarin + Fucoidan	150 or 300 mg/kg 240 mg/kg 150 or 300 mg/kg + 240 mg/kg	After weaning for 35 days	+ ADG in pigs fed 300 mg/kg laminarin-supplemented diets + G:F in pigs fed with diet supplemented solely with 300 mg/kg laminarin or fucoidan (interaction) − FS in pigs fed 150 or 300 mg/kg laminarin-supplemented diets and in pigs fed with diet supplemented solely with fucoidan (interaction)	+ faecal *Lactobacillus* spp. in pigs fed fucoidan-supplemented diets 0 faecal *E. coli*, *Bifidobacterium* spp.	[50]
28-day-old	65% laminarin-rich extract (*Laminaria* spp.)	300 mg/kg	After weaning for 14 days	+ ADG, ADFI 0 diarrhoea score	+ VH in duodenum and jejunum and CD in jejunum − Enterobacteriaceae in caecum + Lactobacillus spp. in colon + butyrate in colon + gene expression of nutrient transporters in small intestine and colon − gene expression of tight junction proteins, mucins and immune markers in small intestine and colon	[51]
35-day-old	Dried seaweed (Ocean Harvest Technology) containing laminarin, fucoidan, alginate, mannitol, fucoxanthin and rhamnose sulphate.	1500 mg/kg	After weaning for 52 days	0 ADG, ADFI, G:F 0 diarrhoea score	− VH in jejunum	[53]
35-day-old	Dried sea weed (Ascophyllum nodosum)	2.5 g/kg 5 g/kg 10 g/kg	After weaning for 28 days	− ADG	ND	[55]
Finisher pigs	Dried seaweed extract (Ascophyllum nodosum) containing laminarin, fucoidan, alginate, mannitol, fucoxanthin and rhamnose sulphate.	3 g/kg 6 g/kg 9 g/kg	After weaning for 28 days	− ADG 0 ADFI, G:F	ND	[56]
28-day-old	65% laminarin-rich extract (*Laminaria* spp.)	300 mg/kg	After weaning for 14 days	+ ADG, ADFI 0 diarrhoea score	− abundance of OTUs assigned to Enterobacteriaceae + abundance of OTUs assigned to the genus Prevotella	[64]
24-day-old	Laminarin (*Laminaria* spp.) Fucoidan (*Laminaria* spp.) Laminarin + Fucoidan	300 mg/kg 240 mg/kg 300 mg/kg + 240 mg/kg	After weaning for 8 days	ND	− Enterobacteriaceae population in pigs offer fucoidan (interaction). − AEEC strains in pigs offer laminarin (interaction). + VH and VH:CD ratio in pigs offered laminarin or fucoidan (interaction). − IL-6, IL-17A and IL-1b mRNA expression in pigs offered laminarin	[65]
24-day-old	Laminarin (*Laminaria* spp.)		After weaning for 8 days	+ ADG and ADFI − diarrhoea score	ND	[66]
24-day-old	Laminarin (*Laminaria* spp.)	0 mg/kg 240 mg/kg ZnO	After weaning for 32 days	+ ADG and G:F, similar effect to ZnO	+ digestibility of GE + the expression of glucose transporters in small intestine compared with the basal diet.	[67]
24-day-old	44% fucoidan-rich extract (*Laminaria* spp.)	0 mg/kg 125 mg/kg 250 mg/kg	After weaning for 14 days	− diarrhoea score 0 ADG, ADFI and G:F	0 effect on VH − abundance of Prevotella and Lachnospiraceae + the abundance of Helicobacter	[68]

+: increase; 0: no effect; −: reduction; N/D: not determined; ADG = average daily gain, ADFI = average daily feed intake, G:F = gain to feed ratio, VH = villous; height, CD = crypt depth, AEEC = attaching effacing *E coli*; GIT = gastrointestinal tract.

## 5. Laminarin

**Figure 1:**
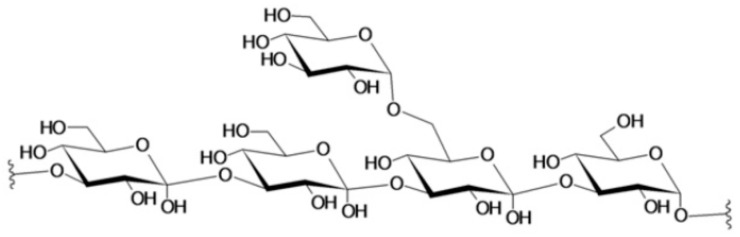
Reported chemical structure of laminarin extracted from *Laminaria digitata* [59].

Laminarins are low molecular weight β-glucans consisting of a linear backbone of (1,3)-β-linked glucopyranose residues with a varying level of β-(1,6)-branching [69] (Figure 1). Water solubility of laminarin depends on the level of branching [70]. Laminarin accumulates in the vacuoles of algal cells during summer and early autumn to support survival and growth during the winter and early spring when it reaches its lowest levels [69,71,72]. In terms of laminarin quantity, *Laminaria hyperborea* and *L. digitata* were reported to have the highest laminarin concentration among the different seaweed species, indicating that *Laminaria* spp. are an important source of this polysaccharide [70].

### 5.1. Antibacterial Activity

Crude laminarin-rich seaweed extracts (*Laminaria* spp.) have exhibited antibacterial activity against *E. coli*, *S.* Typhimurium, *Listeria monocytogenes*, and *Staphylococcus aureus* in vitro [73]. Similar results were observed with purified laminarin (*Laminaria* spp., *Eisenia* spp., *Cystoseira* spp.) from various seaweed species, while it is also evident that laminarin is more effective against Gram-negative than Gram-positive bacteria [74,75]. Dietary supplementation with crude or highly purified laminarin-rich extracts (*Laminaria* spp.) reduced *Enterobacteriaceae* [51,64] and/or the subpopulation of attaching-effacing *Escherichia coli* (AEEC) [65,66] in the caecum and colon of weaned pigs. Similar reductions in ileal and colonic coliform counts were observed in growing [76,77,78] and finishing pigs [79] supplemented with highly purified laminarin-rich extracts (*Laminaria* spp.). In a dextran sodium sulphate (DSS)-induced colitis porcine model, the DSS-challenged pigs supplemented with crude [80] or highly purified [81] laminarin-rich extracts (*Laminaria* spp.) had reduced *Escherichia*/*Shigella* relative abundance and colonic *Enterobacteriaceae* counts, respectively, compared to DSS-challenged control pigs.

### 5.2. Prebiotic Activity

In weaned and grower pig studies, dietary supplementation with crude or highly purified laminarin-rich extracts (*Laminaria* spp.) led to increases and compositional changes in the colonic and faecal *Lactobacillus* spp. populations [51,67,78]. An in-depth investigation of the effects of a crude laminarin-rich extract (*Laminaria* spp.) on the composition of the colonic and caecal microbiota of weaned pigs showed an increased relative abundance in *Prevotella* spp. while its family, *Prevotellaceae*, was positively correlated with improved pig performance [64]. Supplementation with crude or highly purified laminarin-rich extracts (*Laminaria* spp.) also altered the short chain fatty acid (SCFA) production and profile of the gastrointestinal microbiota in pigs [51,77,79], particularly altering butyrate production.

### 5.3. Immunomodulatory Activity

Dietary supplementation with crude or highly purified laminarin-rich extracts (*Laminaria* spp.) exerted an anti-inflammatory effect on the small intestine and colon of weaned and growing pigs evidenced by the decreased expression of proinflammatory cytokine genes including tumour necrosis factor (*TNF*), transforming growth factor beta 1 (*TGFB1*), interleukins *IL1A*, *IL1B*, *IL6*, *IL17A*, and *IL10*, pattern recognition receptors such as toll-like receptor 2 (*TLR2*) and Dectin-1/C-type lectin domain containing 7A (*CLEC7A*), and the transcription factor nuclear factor kappa B subunit 1 (*NFKB1*) [51,65,77]. An immunosuppressive effect due to laminarin was also observed in the colon, more specifically related to the down-regulation of genes associated with the Th17 pathway [82]. The influence of dietary supplementation with highly purified laminarin-rich extracts on the immune response of the porcine intestinal tissue towards a bacterial stimulus was evaluated in an ex vivo LPS challenge model. Here, the colonic tissue of pigs supplemented with highly purified laminarin-rich extracts (*Laminaria* spp.) had higher expression of *IL6* and C-X-C motif chemokine ligand 8 (*CXCL8*) following the LPS challenge, indicating that laminarin might provide improved protection against intestinal bacterial infection via enhanced activation of the immune system [76,77].

### 5.4. Effects of Laminarin-Rich Extracts on Pig GIT Functionality

Several studies have demonstrated the benefits of laminarin-rich extracts as a dietary supplement during the post-weaning period in pigs, as presented in Table 1. Performance parameters such as final bodyweight, daily gain, feed intake, and gain to feed ratio were positively influenced in weaned pigs supplemented with crude or highly purified laminarin-rich extracts (*Laminaria* spp.) [49,50,51,66,67]. Furthermore, dietary supplementation with crude or highly purified laminarin-rich extracts (*Laminaria* spp.) led to improved villus architecture in the small intestine, mainly characterised by increased villus height (VH) and VH: Crypt depth (CD) ratio and increased expression of nutrient transporter genes, indicating enhanced nutrient digestion and absorption, both of which are impaired in the immediate post-weaning period [51,65,67]. Diarrhoea, a common characteristic of weaning stress, was reduced by dietary supplementation with highly purified laminarin-rich extracts (*Laminaria* spp.) as indicated by the lower faecal scores in the supplemented weaned pigs [49,50,65]. In a recent study, Rattigan et al. [52] showed that under hygienic sanitary conditions, laminarin-rich extracts reduced the incidence of diarrhoea in weaned pigs, while under unsanitary conditions, laminarin reduced the incidence of diarrhoea and improved daily gains. Therefore, laminarin-rich extracts seem to be a promising dietary alternative to antibiotic growth promoters and ZnO to alleviate PWD.

## 6. Fucoidan

Fucoidans are a complex and heterogenous group of water-soluble sulphated fucose-rich polysaccharides that contain small quantities of other monosaccharides (e.g., xylose, mannose, galactose, rhamnose, glucose) as well as glucuronic acids and acetyl groups [83]. The backbone structure of fucoidan consists of (1,3)-α-linked fuco-pyranose residues or alternating (1,3)-α- and (1,4)-α-linked fuco-pyranose residues with sulphate groups, occurring mainly at C-2 and C-4 positions and rarely at C-3 [83,84]. The chemical structure of fucoidans vary between different seaweed species. Fucoidan concentration peaks in late autumn/early winter in the various seaweed species of the Fucales order; however, the observed fluctuation is considered relatively small [85]. A higher seasonal variation in fucoidan content was reported in two members of the Laminariales order with summer being most likely the best performing period [86]. Fucose and sulphate content within the total fucoidan also presented monthly variation with potential implications in the bioactivity of the extracted polysaccharide [85]. *Ascophyllum nodosum* is among the fucoidan-rich seaweed species and, thus, is commonly used as a source of this polysaccharide [87,88].

### 6.1. Antibacterial Activity

In an in vitro screening study, crude fucoidan (*Sargassum* spp.) inhibited the growth of several important human bacterial pathogens, though the effect varied between bacterial species [89]. Several studies have reported that depolymerisation improves the antibacterial activity of fucoidan. Lower molecular weight fucoidans (*Laminaria* spp., *Sargassum* spp., *Undaria* spp.) reduced Gram-negative *E. coli, S.* Typhimurium and *Klebsiella pneumoniae*, and Gram-positive *St. aureus* and *Bacillus cereus* in vitro with better efficacy against Gram-negative bacteria, while the crude fucoidans had no effect on the tested bacterial strains [90,91,92,93]. Palanisamy et al. [94] also reported an in vitro antibacterial activity in a fucoidan fraction (*Sargassum* spp.) against Gram-negative bacterial strains comparable to the control antibiotic. The proposed antibacterial mechanisms for low molecular weight fucoidans are: (1) interference with the cell membrane integrity and permeability leading to leakage of cytoplasmic components, cell lysis and death [90,94], and (2) nutrient trapping leading to reduced nutrient availability [91]. The concentration-dependent reduction of *S.* Typhimurium adhesion on a human colonic cell line by fucoidan oligosaccharides indicates that this bioactive may also interfere with pathogen colonisation [95].

Dietary supplementation with a highly purified fucoidan-rich extract (*Laminaria* spp.) reduced the colonic *Enterobacteriaceae* counts in weaned pigs [65]. Furthermore, a crude fucoidan-rich extract (*Laminaria* spp.) was identified as a dietary supplement, promising with regard to its ability to control *S.* Typhimurium infection in growing pigs, as it reduced faecal shedding and colonic and caecal counts of this pathogen [96].

### 6.2. Prebiotic Activity

The ability of fucoidan to modulate the gastrointestinal microbiota and its metabolic products has been the focus of several studies. In vitro, fucoidan (*Fucus* spp., *Sargassum* spp., *A. nodosum*) promoted the growth of *Bifidobacterium* spp. strains and *Lactobacillus delbrueckii* subsp. *bulgaricus*, indicating that this polysaccharide can act as a substrate for these bacterial populations; however, interspecies variation was evident [97,98,99]. In a batch fermentation study with human faeces investigating the prebiotic potential of two fractions of fucoidan (*Laminaria* spp.) varying in molecular weight, the <30 kDa fraction stimulated both *Bifidobacterium* spp. and *Lactobacillus* spp. populations, whereas the >30 kDa fraction increased only *Bifidobacterium* spp. [100]. Both fractions additionally altered the SCFA profile by increasing acetate and butyrate production [100].

Fewer studies within the available literature relate to the effects of dietary fucoidan on the composition of pig GIT microbiota. The most commonly reported change in pigs supplemented with highly purified fucoidan-rich extracts (*Laminaria* spp.) was the increase in *Lactobacillus* spp. in the colon [79] or faeces [49,50]. In a recent study, dietary supplementation with a crude fucoidan-rich extract (*A. nodosum*) altered the composition of the caecal microbiota, including increases in members of the Bacteroidetes phylum, and increased propionate and butyrate production in the colon of weaned pigs [68].

### 6.3. Immunomodulatory Activity

To gain a better insight in the immunomodulatory activity of fucoidan, Zhang et al. [101] conducted a series of in vitro and in vivo experiments using fucoidans isolated from *A. nodosum*, *Fucus vesiculosus*, *Macrocystis pyrifera*, and *Undaria pinnatifida*. All fucoidans delayed apoptosis and stimulated the production of the proinflammatory cytokines IL6, CXCL8, and TNFα in human neutrophils [101]. Furthermore, these fucoidans were identified as potent adjuvants of cellular and humoral immune responses, due to their involvement in the activation, maturation, and functionality of Natural Killer (NK) cells, dendritic cells, T cells, and antibody production in mice [101]. However, variation in the bioactivity of the fucoidans from different seaweed species was also evident [101]. Fucoidan most likely interacts with the immune cells via pattern recognition receptors scavenger receptor class A (SR-A), TLR2, and TLR4 [102,103]. Fucoidan is also associated with reduced inflammation following bacterial stimulus, e.g., LPS. A crude fucoidan-rich extract (*Sargassum* spp.) reduced the production and expression of proinflammatory markers such as nitric oxide (NO), TNFα, IL1β, and IL6 proteins and *IL1B*, inducible NO synthase (*iNOS*) and cyclooxygenase-2 (*COX-2*) genes, and the expression of the transcriptional factor *NF-κB* in murine macrophages following a LPS challenge [104]. The anti-inflammatory activity of fucoidan was also observed in a series of similar studies on LPS-challenged murine macrophages, whereby fucoidans from different seaweed species (*Ecklonia cava*, *L. japonica*) were used [105,106]. Interestingly, molecular weight and sulfation level were considered important determinants of the immunomodulatory activity of fucoidan [105,106]. These findings suggest that the effect of fucoidan on the immune system probably depends on its seaweed source, structure, and composition, and the state of inflammation in the host.

The anti-inflammatory potential of fucoidan was observed in *S.* Typhimurium infection and DSS-induced colitis models in pigs. Dietary supplementation with a crude fucoidan-rich extract (*Laminaria* spp.) reduced the expression of several inflammatory markers, namely *TNF, IL6*, *IL22*, and regenerating family member 3 gamma (*REG3G*) in the colon of *S.* Typhimurium-infected pigs [96]. Furthermore, the increased *IL6* expression in the DSS-challenged pigs was suppressed by dietary supplementation with a highly purified fucoidan-rich extract (*Laminaria* spp.) [81].

### 6.4. Effects of Fucoidan-Rich Extracts on Pig Performance and GIT Functionality

The effects of dietary fucoidan-rich extracts on performance parameters in pigs are less pronounced and inconsistent across studies. In weaned pigs, dietary supplementation with crude or highly-purified fucoidan-rich extracts (*Laminaria* spp., *A. nodosum*) had no effect on final body weight, daily gain, feed intake, and food conversion ratio [49,67,68], although increases in feed efficiency has been previously reported [50]. Improved performance was observed in growing pigs supplemented with a crude fucoidan-rich (*Laminaria* spp.) extract in a study with an experimental *S.* Typhimurium challenge [96]. Variable results regarding villus architecture and expression of nutrient transporters genes in the small intestine of weaned pigs supplemented with crude or highly purified fucoidan-rich extracts (*Laminaria* spp., *A. nodosum*) were also evident across studies [65,67,68]. Dietary supplementation with crude or highly purified fucoidan-rich extracts (*Laminaria* spp., *A. nodosum*) was additionally found to reduce faecal scores in weaned pigs [50,65,68]. The improved faecal consistency coupled with the enhanced performance under challenging conditions warrant further research into the potential of fucoidan-rich extracts as a dietary supplement to prevent or control PWD in pigs.

## 7. Laminarin and Fucoidan Interaction

The supplementation of intact seaweed has been less successful in the immediate post-weaned pig diet [54,55,107], as summarized in Table 1. This is probably due to a negative interaction between laminarin, fucoidan, other non-digestible polysaccharides, polyphenols, and minerals on digestive health and performance in the post-weaned pig. For example, in a study by Walsh et al. [65], supplementation with fucoidan alone reduced *Enterobacteriaceae*, but when combined with laminarin this effect was not observed. Similarly, laminarin supplementation resulted in a reduction in AEEC strains, while pigs offered either laminarin or fucoidan had increased VH and VH:CD in the duodenum, but when offered in combination these effects were not observed [65]. Similarly, Lynch et al. [108] observed a reduction in *Enterobacteriaceae* and an increase in butyric acid in the colon of laminarin supplemented pigs, but again these effects were not observed when laminarin and fucoidan were supplemented together. In the study by McDonnell et al. [49], pigs fed fucoidan or laminarin alone had improved daily gain compared with pigs fed the basal diet, but this positive effect was not observed when the polysaccharides were fed in combination. In the same study, pigs supplemented with fucoidan had increased *lactobacilli* populations but when combined with laminarin this benefit was lost [49]. Supplementation with laminarin increased the coefficient of total tract apparent digestibility of gross energy and increased the gene expression of nutrient transporters SGLT1, GLUT1, and GLUT2 compared with the basal diet, but the effect on these variables was lost when laminarin and fucoidan were combined [67]. These results suggest that laminarin and fucoidan have differing modes of action and their effects are not synergistic, leading to the less successful supplementation of whole seaweeds in the immediate post-weaned pig diet. In summary, the purest forms of laminarin and fucoidan extracted individually from macroalgae appear to have the most benefit in terms of improvements in GIT health compared with intact macroalgae, as intact combinations of laminarin and fucoidan are likely to complex together and thus are less effective.

Traditional methods of laminarin and fucoidan extraction are energy intensive, time consuming, utilise large volume of solvents, and result in poor yield, whereas new extraction techniques such as hydrothermal-assisted extraction are low cost, easy to use, and environmentally friendly methodologies that can be easily scaled-up for the industrial production of laminarin and fucoidan [109].

## 8. Feeding Seaweed Extracts to the Pregnant and Lactating Sow

Neonatal piglets are rapidly colonised during birth and suckling with microorganisms from the vaginal and faecal microbiota of the sow as well as the environment. There is evidence that when neonatal pigs are less exposed to potentially pathogenic bacteria, they have a lower chance of developing PWD [110,111,112]. Supplementing pregnant sow diets during late gestation with seaweed extracts containing laminarin and fucoidan reduced the *Enterobacteriaceae* population in the sow’s faeces, while also reducing colonic *Escherichia coli* numbers in the piglets at weaning [113,114]. This indicates that modifying the microflora of the sow has the potential to influence the microbial profile of her offspring.

The immunoglobulin profile of the colostrum/milk that is ingested by the piglet has the potential to deliver antimicrobial effects [114] and immune enhancing properties [115,116]. Supplementing sow diets during late gestation with seaweed extracts containing laminarin and fucoidan increased piglet serum IgG concentration on day 14 of lactation [113], while piglets suckling these seaweed extracts supplemented sows also had improved leukocyte phagocytosis capacity [115]. Improved resistance to infection and reduced pathogen shedding post-weaning were also observed in piglets suckling laminarin and fucoidan supplemented sows, following an ETEC challenge [111] and a *S.* Typhimurium challenge [117]. The purity of laminarin and fucoidan does not appear to be as important in the lactating sow diet, as the sow seems more capable of utilising the combination of laminarin and fucoidan in the diet than the younger pig [111,113].

## 9. Chitin and Its Derivatives

Both chitin, chitosan, and their derivatives have attracted considerable interest due to their biological activities, including antimicrobial, antitumour, immune stimulatory effects, and the acceleration of wound healing [118,119]. Chitin and chitosan are biopolymers composed of glucosamine and N-acetylated glucosamine (2-acetylamino-2-deoxy-D-glucopyranose) units linked by β (1–4) glycosidic bonds [120]. Chitosan is produced via chemical or enzymatic modification of chitin through removing the acetyl group from the chitin, a process called de-acetylation.

### 9.1. Antibacterial Effects of Chitosan and COS

There is a lot of variation in the literature on the antibacterial properties of chitosan and chito-oligosacharide (COS), which is partly due to the widely different molecular weight (MW) used across studies [118,121]. In pigs, supplementation with 5–10 kDa and 10–50 kDa COS increased lactic acid bacteria (LAB), while 50–100 kDa reduced LAB, and all molecular weights were shown to reduce *E. coli* in the weaner period [112]. An increase in lactobacilli counts were also observed on day 14 and 21 post-weaning and a reduction in faecal *E. coli* numbers in pigs supplemented with COS [122,123]. COS supplementation was also shown to reduce *E. coli* in the caecum of weaned pigs [124]. COS has been shown to prevent the adhesion of some strains of enteropathogenic *E. coli* to intestinal cells in vitro [125]. N-acetylglucosamine is a component of many mammalian glycoconjugates, particularly of mucins [126] which are involved in the prevention of bacterial binding to the intestinal surface, thus the N-acetylglucosamine in COS may bind with certain bacteria and prevent their attachment to the intestinal epithelium [123,124,125]. COS may also act as a substrate for the growth of beneficial bacterial species [120,127] and may lead to reduced intestinal pH [128], thereby reducing the proliferation of pathogenic bacteria [124].

### 9.2. Effects of COS on Growth Performance

COS supplementation of varying molecular weights improved daily gain, feed efficiency, and reduced diarrhoea scores in the weaner period [122]. Supplementation of COS to weaned pigs challenged with *E. coli* K88 improved faecal scores but did not improve daily gain or feed efficiency [124]. However, COS supplementation increased VH:CD compared to the unsupplemented challenged pigs [124]. Similarly, an increase in villus height and VH:CD was observed in the jejunum of COS-supplemented pigs [129]. Plasma levels of insulin-like growth factor 1 (IGF-1) were increased in COS-supplemented pigs 48 h post-infection with *E. coli* K88 and remained greater than that of the un-supplemented challenged pigs [114]. Supplementation with 250 mg/kg COS led to improved growth and feed efficiency through increased plasma growth hormone and IGF-1 levels in early weaned pigs [129]. The inclusion of 100 and 200 mg/kg COS increased daily gain and feed efficiency, with the 200mg/kg inclusion rate achieving similar results as chlortetracycline supplementation in weaned pigs [123]. Inclusion of 200mg/kg COS also improved the apparent digestibility of gross energy, crude protein, calcium, and phosphorous, and reduced the incidence of diarrhoea compared with the control group [123]. COS supplementation also improved jejunal and ileal morphology compared with the control, thus the improvements in daily gain may be related to increased feed intake, enhanced intestinal morphology, and improved nutrient digestibility [123]. Therefore, COS have the potential to be a very promising dietary alternative to antibiotic growth promoters and ZnO in alleviating PWD.

## 10. Conclusions

Dietary interventions are a promising strategy to alleviate or prevent dysbiosis and the associated intestinal diseases and disorders that negatively impact on performance and health in post-weaned pigs. The increasing concern around AMR and environmental contamination has led to increasing pressure for alternative dietary supplements to enhance post-weaning growth performance and control PWD in piglets instead of AGPs, antimicrobials, and ZnO. Recent research has proven that the inclusion of the marine derived bio-actives, including chitosan, COS, fucoidan, and laminarin, could affect the pig’s intestinal health and growth performance in the post-weaning phase. These supplements could therefore support the intestinal immune system, microbiology, and morphology of the post-weaned pig, leading to enhanced growth performance. Indeed, these supplements could be suitable substitutes for in-feed antimicrobials and ZnO. Several studies have also shown the positive effects of feeding sows SWE extracts on the neonatal piglet by enhancing the immune response and reducing shedding of pathogenic bacteria. The supplementation of intact seaweed has been less successful in the immediate post-weaned pig diet, while supplementation with the purest forms of laminarin and fucoidan extracted individually from macroalgae appears to have the most benefit in terms of improvements in GIT health. However, the extraction methodologies and conditions used to extract these polysaccharides, along with varieties of seaweed used, are an important contributing factor to the quantitative, structural, and functional variability of seaweed polysaccharides.

## Data Availability

Not applicable.
